# Suppression of Graft Regeneration, Not Ischemia/Reperfusion Injury, Is the Primary Cause of Small-for-Size Syndrome after Partial Liver Transplantation in Mice

**DOI:** 10.1371/journal.pone.0093636

**Published:** 2014-04-07

**Authors:** Ning Pan, Xiangwei Lv, Rui Liang, Liming Wang, Qinlong Liu

**Affiliations:** 1 Department of General Surgery, The Second Affiliated Hospital of Dalian Medical University, Dalian, Liaoning Province, China; 2 Department of Anesthesiology, The Second Affiliated Hospital of Dalian Medical University, Dalian, Liaoning Province, China; Université Paris Sud, France

## Abstract

**Background:**

Ischemia/reperfusion injury (IRI) is commonly considered to play a crucial role in the pathogenesis of small-for-size syndrome (SFSS) after liver transplantation. Rapid regeneration is also considered essential for the survival of SFS grafts.

**Methods:**

Mouse models of full-size orthotopic liver transplantation, 50% partial liver transplantation and 30% partial liver transplantation were established. Survival rate and serum alanine aminotransferase were observed. IRI was assessed by hepatic pathologic alterations, apoptosis and necrosis. Regeneration response was detected by mitotic index, BrdU incorporation and PCNA, Cyclin D1 and Cyclin E expression. The expression of mTOR, AKT, ERK, JNK2 and p70S6K, also involved in regeneration signaling pathways, were analyzed as well.

**Results:**

30% partial liver graft resulted in a significantly low 7-day survival rate (P = 0.002) with no marked difference in tissue injury compared with the 50% partial graft group. Serum alanine aminotransferase levels were not significantly different between partial transplantation and full-size transplantation. Western blot analysis of caspase-3 and TUNEL staining also indicated no significant difference in apoptosis response between 30% partial transplantation and half-size or full-size transplantation (P = 0.436, P = 0.113, respectively). However, liver regeneration response indicators, mitotic index (P<0.0001) and BrdU (P = 0.0022), were markedly lower in 30% LTx compared with 50% LTx. Suppressed expression of PCNA, cyclin D1, cyclin E, mTOR, JNK2, AKT, ERK and p70S6K was also detected by western blot.

**Conclusions:**

Liver regeneration is markedly suppressed in SFSS, and is more likely the primary cause of SFSS, rather than ischemia/reperfusion injury. Therapy for recovering graft regeneration could be a potentially important strategy to reduce the incidence of SFSS.

## Introduction

The widespread application of liver transplantation for end-stage liver diseases, a shortage of deceased donors and advancements in modern techniques of hepatectomy have made living donor liver transplantation (LDLT) a routine procedure [1]. Moreover, based on the regeneration potential of hepatocytes, the use of partial liver transplantation has increased rapidly in recent years, and has dramatically alleviated mortality on the waiting list [2]. The application of smaller grafts would be a revolution in transplantation, however, in clinical practice, a major concern is the adequacy of recipient graft volume while retaining a sufficient remnant liver volume within the donor [3].

In adult-to-adult living donor and cadaveric split liver transplantation (LT), a graft to recipient weight ratio (GRWR) of less than 0.8–1.0%, corresponding to less than 30%–50% of standard liver volume (SLV), has been used to define small-for-size (SFS) grafts [4], [5]. Although it was reported in an early study on LDLT that transplanted grafts approached expected/standard liver volumes in time, regardless of graft size mismatching [6], difficulties related to SFS grafts have emerged with the expansion of LDLT. SFS graft recipients appear to have a greater risk of poor prognosis, including coagulopathy, ascites, prolonged cholestasis, encephalopathy, pulmonary and renal failure, and reduced graft survival. This ill-defined clinical picture has been considered to be primarily linked to insufficient graft size and has been termed “small-for-size-syndrome” (SFSS)[7].

While SFSS is agreed to be a distinct disease entity, the direct mechanisms remain unclear, and complex elements and factors are involved. Ischemia/reperfusion injury (IRI), one of the several non-immunological elements closely associated with LT outcome, has been extensively studied and is known to impair remnant liver and small-for-size graft regeneration, and to contribute to graft dysfunction following LT [8], [9], [10]. Paradoxically, ischemic preconditioning (IPC), a strategy that provides protection against IRI and improves regeneration capacity [11], [12], [13], has been shown to significantly worsen the extent of graft injury and hinder hepatic regeneration in SFS LTx models [14]. These contradictory findings suggest that the primary cause of SFSS may not be IRI.

Liver regeneration is a complex process involving multiple cytokines and growth factors (TNF-α,IL-6,HGF) critical for survival and rapid recovery following hepatectomy and LTx. Regeneration has been shown to be markedly inhibited after >70% hepatectomy and SFS liver graft transplantation [15], [16], [17], [18], leading to compromised liver function and graft loss. To elucidate possible causes of hepatic graft failure in SFSLT, we investigated liver regeneration responses and ischemia/reperfusion injury after transplantation with grafts of varying size.

## Materials and Methods

### Ethic Statement

This study was carried out in strict accordance with the recommendations in the Guide for the Care and Use of Laboratory Animals of the National Institutes of Health. The protocol was approved by the Committee on the Ethics of Animal Experiments of the Dalian Medical University, China (Permit Number: SYXK (Liao) 2008–0002). All surgery was performed under isoflurane anesthesia, and every effort was made to minimize suffering. After transplantation, all recipients were warmed with a heating pad and had free access to standard laboratory chow and tap water adlibitum. Fentanyl was used 24 hours after surgery. Mouse condition was monitored every hour in the daytime and every 6 hours at night. Mice were under isoflurane anesthesia again before collecting blood and liver samples for clinical chemistry, histology and immunohistochemistry, and then sacrificed by cervical dislocation under anesthesia. For survival studies, mice in markedly poor condition were considered to have failed to survive from transplantation. These mice were then put into coma with carbon dioxide and humanely euthanized by cervical dislocation. Mice which had lived for more than 7 days after transplantation were considered survivors.

### Animals

Male C57BL/6 mice (10–12 weeks, 27–30 g) were used as both donors and recipients. Mice were housed in a standard animal laboratory with free activity and access to food and water, and kept under constant environmental conditions with a 12∶12-h light:dark cycle. Mice were fasted for 12 h before surgery.

### Study Design and Partial Liver Transplantation

Mice were randomly divided into four groups: sham operation, full-size graft (FSG), 50%-size graft (HSG) and 30%-size graft (TSG) liver transplantation groups. Group sizes were 8–10 each in the FSG, HSG and TSG group, respectively. Orthotopic liver transplantation (LT) was performed under isoflurane anesthesia and all efforts were made to minimize suffering [19], [20]. Following the model developed by Tian, a classic model in the study of liver regeneration, the left lateral lobe, the anterior and posterior caudate lobes and the median lobe, or the left portion of the median lobe, were removed after ligation with 5–0 silk suture resulting in reduction of liver mass by 30% (30%-size grafts, TSG) and 50% (half-size grafts, HSG) [15], [16]. Full-size grafts (FSG), HSG and TSG were explanted and stored in UW solution (University of Wisconsin solution) for 4 hours, as per standard clinical protocol [21]. All surgery was performed under isoflurane anesthesia, and every effort was made to minimize suffering. Survival was monitored for 7 days following surgery. Mice that had lived for more than 7 days after transplantation were considered survivors.

### Clinical Chemistry, Histology and Immunohistochemistry

Blood was collected from the inferior vena cava and livers were recovered. Serum alanine transaminase (ALT) was measured using analytical kits from Pointe Scientific (Uncoln Park, MI) [22]. Histology was examined after hematoxylin–eosin (H&E) staining. Apoptosis was assessed by terminal deoxynucleotidyl transferase dUTP nick-end labeling (TUNEL) using an In Situ Cell Death Detection Kit. BrdU incorporation into liver sections was determined immunohistochemically and quantified as described previously [22]. Mitotic cells were counted in a blinded manner in 10 random fields in H&E stained liver sections using a 40× objective lens.

### Statistical Analysis

Data are presented as mean ± SEM. Groups were compared using a Kaplan–Meier test and ANOVA plus Student–Newman–Keuls, as appropriate. Numbers of animals were 8 to 10 per group in the survival experiment and 6 per group for all others, as indicated in figure legends. Differences were considered significant at p<0.05.

## Results

### 30% Partial Liver Graft Resulted in a Significantly Low 7-day Survival Rate

**Figure 1:**
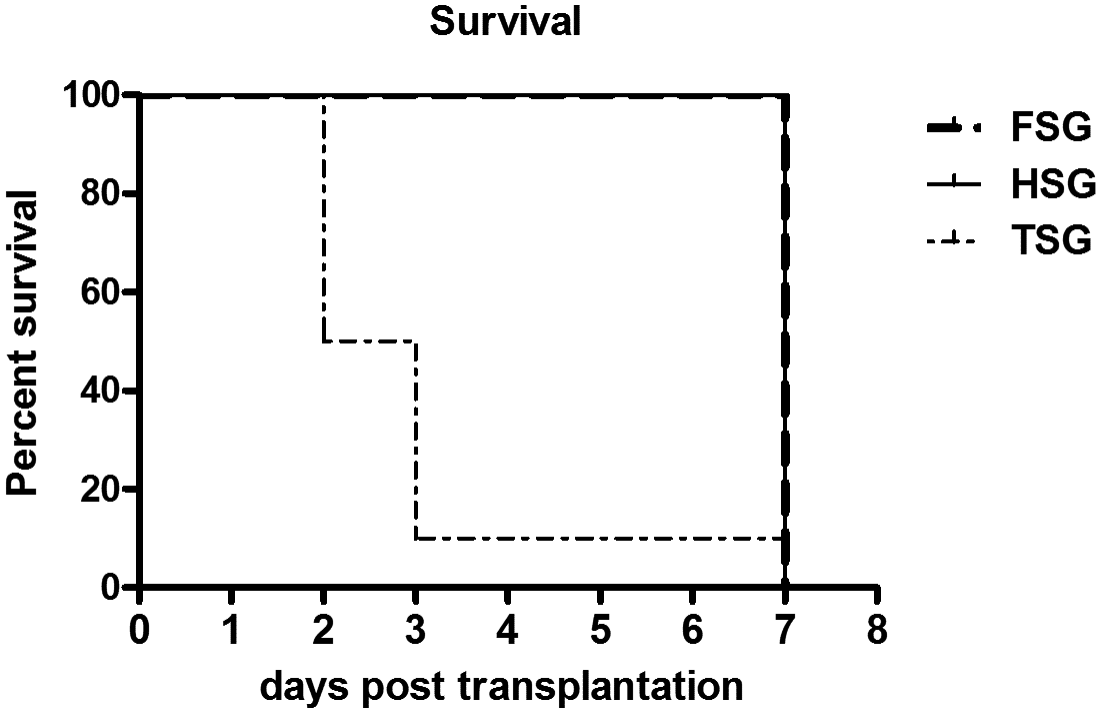
Decreased survival rate after transplantation of small-for-size liver grafts. Mice were observed 7 days postoperatively for survival. Group sizeswere 8–10 each in the FSG, HSG and TSG groups, respectively. P<0.05 by the Kaplan-Meier test. At 48h post-transplantation, when the samples were taken, the 1-day survival rate of TSG was 100%.

Seven-day survival rates in each group were 100% (FSG), 100% (HSG) and 10% (TSG), with a significant difference between the TSG group reduced survival rate compared with the other groups (P = 0.002), as shown in figure 1.

### Serum Alanine Aminotransferase (ALT) Levels were not Significantly Different between Partial and Full-size Transplantation

**Figure 2:**
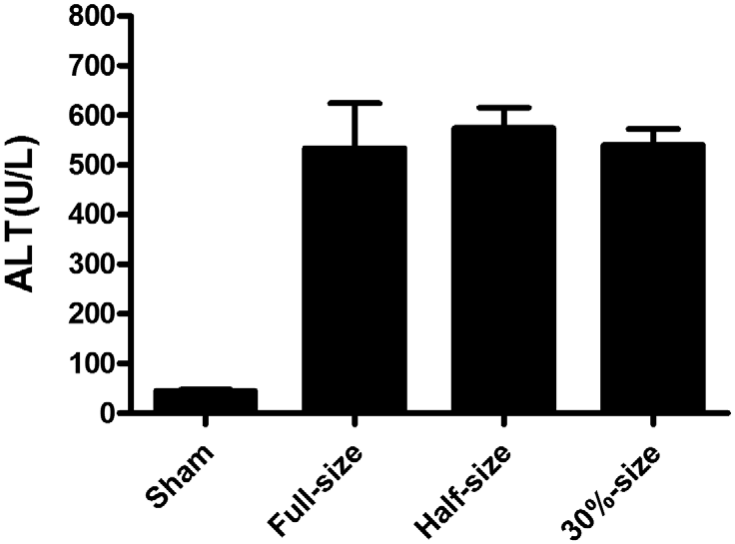
ALT levels in mice receiving liver grafts of various sizes. Before sacrificing the mice for histology, blood was collected for determination of serum alanine aminotransferase levels. Means + SD of six mice each are given.

Serum ALT levels in each group were 45.00±2.828 U/L (Sham), 534.3±36.65 U/L (FSG), 574.2±41.28 U/L (HSG) and 540.0±32.06 U/L (TSG). As shown in figure 2, there were no statistical differences between partial and full-size transplantation groups or between the TSG and HSG groups.

### H&E Staining for Mitotic Index (MI) Showed no Marked Differences between the HSG and TSG Groups

**Figure 3:**
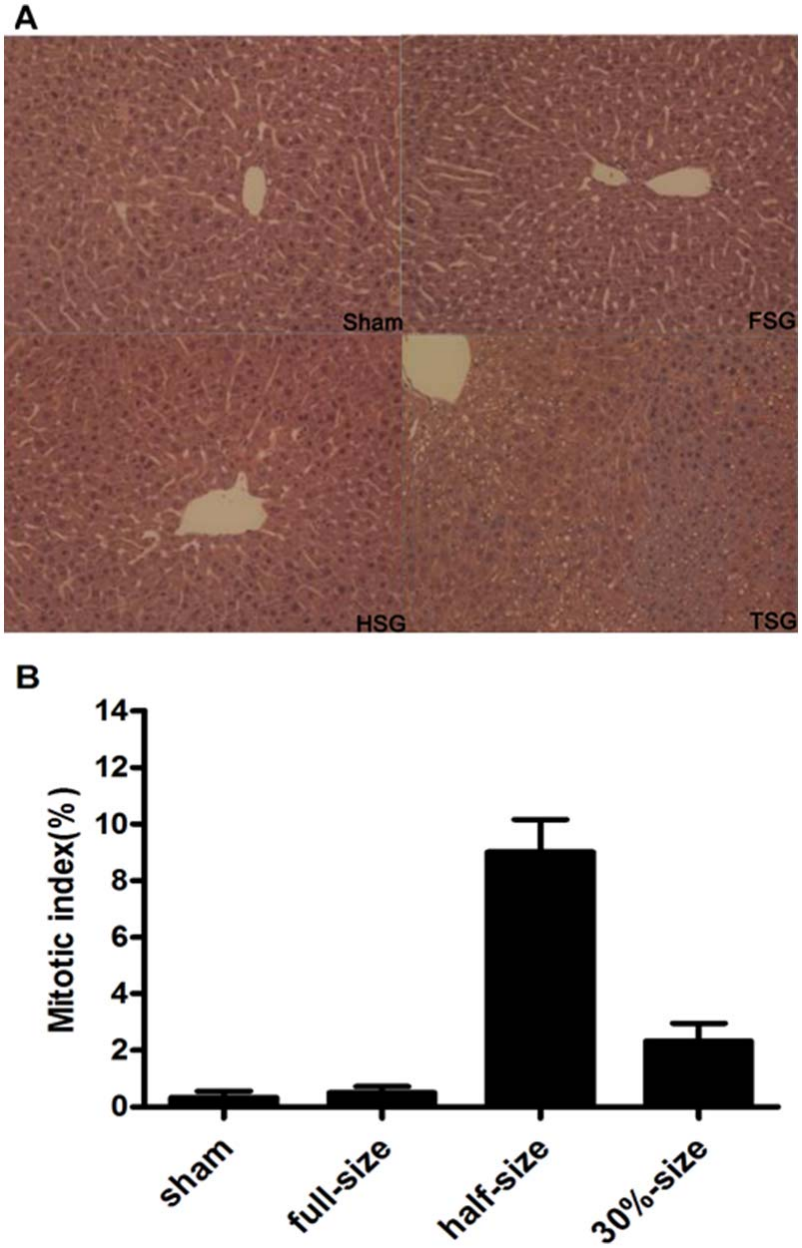
H&E staining for Mitotic Index. Sham-operation and varying size graft LTx were performed as described in METHODS. Livers were harvested at 48 h after surgery and stained with hematoxylin and eosin to assess necrosis. Representative images are shown (bar = 100 μm). Ten random fields per slide were captured in a blinded manner using a 10× objective lens. Mitotic hepatocytes were counted in 10 random fields per H&E stained liver section and plotted in figure 3.

Mitotic cells were minimal in sham-operated livers and in the FSG group (figure 3A). Post-transplantation, the mitotic index increased to 8% in the HSG group, with no increase in the TSG group (figure 3B).

### Caspase-3 Expression as Indicated by Western Blot and TUNEL Staining Showed that there was no Difference in Apoptosis in the TSG and HSG Groups at 48 Hours following Transplantation

**Figure 4:**
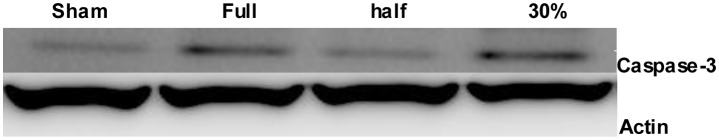
Western Blot for expression of caspase-3. Alterations of caspase-3 expression after liver transplantation. Livers were collected 48 h post-transplantation. Caspase-3 was detected by immunoblotting. Representative gels are shown.

**Figure 5:**
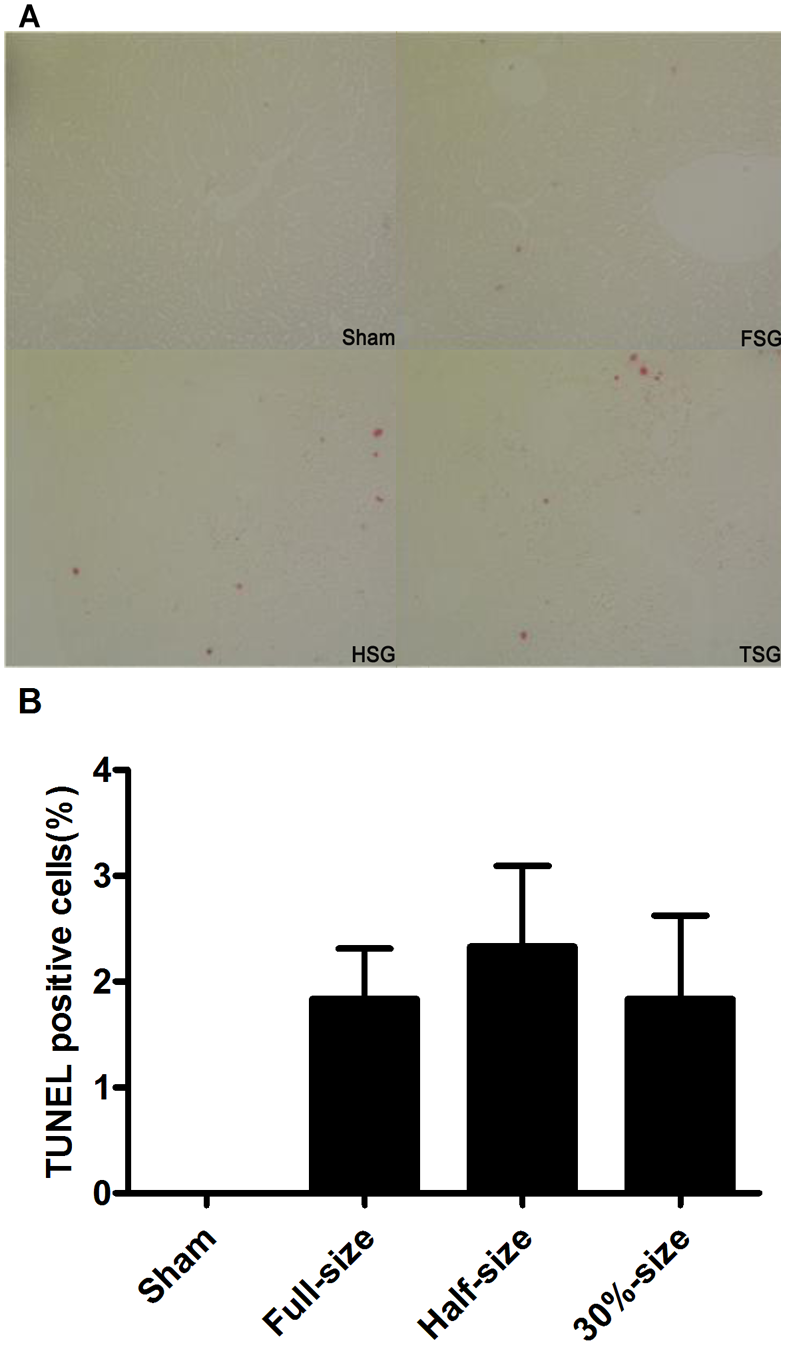
TUNEL staining for liver grafts 48 hours after implantation. Liver grafts were fixed and sectioned, and apoptotic cells were detected by immunohistochemical DNA strand break labeling. Ten fields were captured at random under a 10× objective lens. TUNEL-positive and -negative cells were counted.

Caspase-3 expression was marginally elevated in the FSG and TSG groups at 2 days post-transplantation, but was not significantly elevated in HSG and TSG groups (figure 4). Few TUNEL-positive cells were found (figure 5A) in liver tissue post-sham operation and only 1.833±0.4773 positive cells/HPF were observed in the FSG group (figure 5B). TUNEL increased to 2.333±0.7601 cells/HPF in the HSG group and decreased to 1.833±0.7923 cells/HPF in the TSG group.

### 5-bromo-2′-deoxyuridine (Brdu) Analysis Showed that Hepatocyte Proliferation and DNA Synthesis were Markedly Suppressed in the TSG Group Compared with the HSG Group

**Figure 6:**
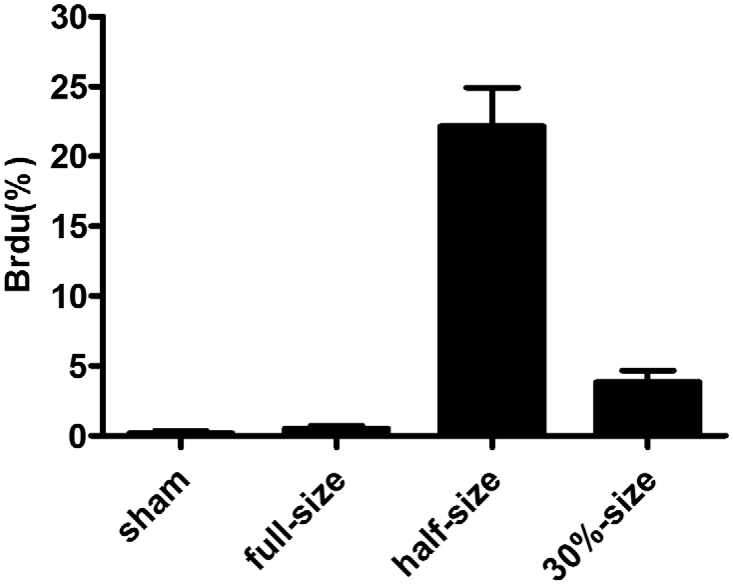
Detection of BrdU-labeled hepatocytes. Liver grafts were recovered 48-positive and negative cells in 10 random fields per slide were counted under a microscope with a 40× objective lens. The percentage of BrdU-labeled hepatocytesis plotted in figure 6.

BrdU labeling was barely detectable 48 h after sham operation in the FSG group (figure 6), while it increased to ∼23% in the HSG group and was only 1% in the TSG group.

### Liver Graft Hepatocyte PCNA, Cyclin E, Cyclin D1, Phospho-Akt, Phospho-mTOR, Phospho-p70S6 Kinase (p-p70S6k), Phospho- ERK and Phospho-JNK2 Expression

**Figure 7:**
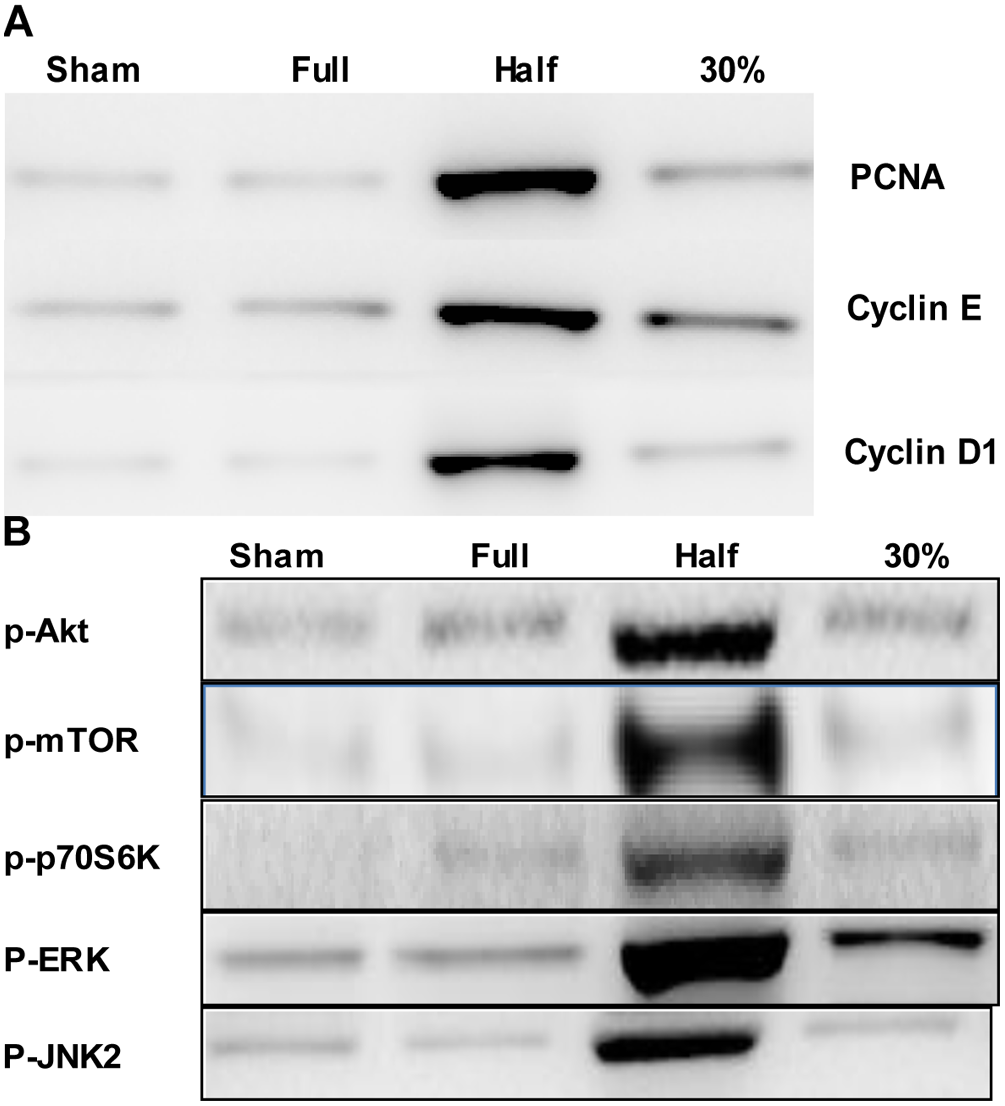
Western Blot results of the four groups. **A:** Livers were recovered at 48-transplantation. PCNA, cyclin D1 (CyD1) and cyclin E (Cy E) were detected by immunoblotting. Representative gels of 4 livers/group are shown. **B:**Alterations of EGFR downstream signaling molecules after liver transplantation. Livers were collected 48 h post-transplantation. Phospho-Akt, phospho-mTOR, phospho-p70S6 kinase (p-p70S6K), phospho-ERK, phospho-JNK and actin were detected by immunoblotting. Representative gels are shown.

PCNA, Cyclin E and Cyclin D1 were barely detectable in sham-operated livers and did not increase in the FSG group (figure 7A). In the HSG group, expression increased markedly at 48 h post-transplantation, while the increases in the TSG group were slight. The expression of EGFR downstream signaling molecules also displayed similar differences (figure 7B).

## Discussion

Small-for-size liver transplantation reduces survival and increases complication rates. With the extensive use of living donor liver grafts in adult patients, controversy over small-for-size syndrome has escalated in recent years and the mechanisms of small-for-size graft failure are still unclear. Complications have been explained as the result of a decreased liver mass failing to meet the metabolic demands of the recipient. However, this explanation does not entirely explain subsequent graft failure and dysfunction. Complex pathogenic aspects are involved, including pre-existing liver disease, latent disease (steatosis, ethanol injury), regeneration, vascular inflow (portal hyperperfusion, arterial hypoperfusion), age, health status, cirrhosis, anatomic variations in vascular structure, warm/cold ischemia and vascular reconstruction [7], [23], [24]. Among these factors, suppression of liver regeneration and increased graft injury and necrosis have been shown to be closely associated with small-for-size graft failure [2], [12], [13], [18], [23], [25].

The results of this study indicate that suppressed liver regeneration is a more likely primary factor in small-graft failure, rather than ischemia/reperfusion injury. Analysis was carried out 48 hours post-operation with 30%-size liver grafts not showing marked differences in structural damage compared with half-size grafts. Graft function and survival rate were severely impaired in recipients of 30%-size grafts compared to those of half-size and full-size grafts. This graft dysfunction and failure post-SFS graft transplantation is consistent with results from previous studies [26], [27].

Alanine aminotransferase (ALT) has been routinely used as a sensitive marker for evaluating the extent of hepatocellular injury and correlates with mitochondrial swelling and hepatocyte apoptosis [12]. According to Tian et al, serum ALT reached 777±165 U/L at 2 hours post-OLT operation in C57BL/6 mice receiving 45% ∼50% size grafts, and reduced to 530 U/L at 48 hours, while serum ALT in mice receiving 30% size grafts was much higher (>2000U/L) at 2 days post-operation [15]. In the present study, there was no significant difference in serum ALT between 30%- and half-size grafts at 48 hours post-OLT operation (figure2).

Tian et al also carried out histologic analysis of OLT grafts in all four groups at 48 hours post-operation and found typical structure injury, including microvesicular steatosis and small necrotic foci containing neutrophils around interlobular bile ducts and within bile duct epithelia in HSG and TSG [15]. In other previous studies using rat or C57BL/6 mouse models for orthotopic liver transplantation, TSG demonstrated greater histological damage post-transplantation than full-size and half-size grafts [16], [18]. Our data showed that although the TSG group displayed diffuse microvesicular steatosis, there was little difference in structural injury between the HSG and TSG groups.

We also evaluated hepatocellular apoptosis to assess the extent of IRI. Apoptosis is activated during the early phase of reperfusion following post-transplantation liver ischemia in animals. According to some observations of hepatic IR, 50%–70% of endothelial cells and 40%–60% of hepatocytes undergo apoptosis during reperfusion. A high percentage of apoptotic hepatocytes have also been identified in human liver allografts [28].

Caspase-3 is a key mediator of apoptotic cell death and its activation indicates that the apoptotic pathway has progressed to an irreversible stage. Qiuxia Fu’s group recently established a new mouse model, using bioluminescence imaging to monitor caspase-3 activity *in vivo*, thus reflecting liver apoptosis during inflammatory and infectious events [27]. Furthermore, liver injury following ischemia/reperfusion can be prevented with caspase inhibition [29]. Our western blot data (figure 4) showed that caspase-3 expression was nearly undetectable after sham operation and half-size-graft transplantation at 2 days post-transplantation. Caspase-3 expression was not significantly elevated in HSG and TSG groups, indicating similarity in the I/R injury apoptosis response.

As another measure of apoptosis, terminal deoxynucleotidyl transferase-mediated dUTP nick-end labeling (TUNEL) was performed on tissue sections to assess double-stranded DNA breaks characteristic of apoptosis [19](figure 5A). Consistent with caspase-3 expression, TUNEL staining also indicated that the IRI apoptosis response was not different between HSG and TSG transplantation (P = 0.6586). Similar results were also found by Zhong et al. In their rat model receiving partial liver grafts, apoptosis was mild as assessed by TUNEL, with only 0.5% positive cells in full-size grafts and 0.3% in 30%-size grafts at 5 hours post-transplantation, with no further increase after 18 to 38 h [17]. Taken together, this indicates that apoptosis due to IRI is mild in partial liver transplantation and unlikely to play a prominent role in impaired survival and proliferation of SFS grafts.

Regeneration is critically required in the setting of LDLT, especially in small-for-size LT, because small-for-size grafts require vigorous and immediate hepatocyte proliferation. According to some studies, a remnant liver of 10% can be enough for survival in rats, but more volume is required for transplantation [5]. It has been shown that three months after partial liver transplantation (50%∼60% size) liver volume slightly exceeds 100% of the standard liver volume (SLV) in recipients, but reaches only about 80% of the SLV in donors [30], an indication of the robust regeneration in grafts due to increased stimulus. However, it was shown that the graft increase ratio becomes much lower in 30% partial LT compared to 50% partial LT (19.9%±3.6% versus 69.8±9.7% at 3 days post-transplantation) [18], evidence of the markedly suppressed regeneration ability that can occur in SFS LT.

The study and application of ischemic preconditioning (IPC), a strategy that has demonstrated protection against IRI and improves regeneration capacity, has been proven effective in reducing hepatic IRI and stimulating liver regeneration, however, it significantly enhances the extent of graft injury and hinders hepatic regeneration in SFS LTx models [14]. Therefore, it is supposed that a shift in regeneration ability is likely a more prominent factor than IRI in liver graft dysfunction and failure following small-for-size transplantation.

To evaluate hepatocyte proliferation, mitotic index (MI) was determined (figure 6a). No increase in TSG suggested that liver regeneration was suppressed in TSG, which we have previously reported [20].

To detect cells synthesizing DNA, 5-bromo-2′-deoxyuridine (BrdU) labeling was performed. BrdU incorporation into DNA is a specific marker for the S-phase of the cell cycle. Hepatic proliferative response was evaluated by the expression of PCNA, cyclin D1 (CyD1) and cyclin E (CyE). Proliferative cell nuclear antigen (PCNA) is a cell-cycle nuclear protein expressed in late G1 and throughout the S-phase of mitosis, and is a processing factor for DNA polymerase δ [31]. PCNA was barely detectable in sham-operated livers and did not increase in FSG, an indication of minimal regeneration activity. In HSG, PCNA expression increased markedly at 48 h post-transplantation, however, the increase in TSG was slight (figure 7A).

In liver regeneration, cell cycle progression includes two steps, the first step involves the transition of hepatocytes from G0 to G1, and the second involves entry into and progression through S phase [32]. CyD1 and CyE timing and expression patterns were first reported in 2007 and were used to monitor the two steps of hepatocellular cell cycle progression [14].

In hepatocytes, CyD1 plays an important role in driving proliferation and CyD1 over expression was shown to be sufficient to promote hepatocyte replication and liver growth *in vivo* [33]. The JNK/c-Jun pathway regulates CyD1 gene expression and the CyD1 promoter contains an c-Jun-activated protein 1 (AP-1) site. AP-1, as well as NF-κB, STAT3 and C/EBPβ, are important transcription factors participating in the priming phase of liver regeneration, making hepatocytes responsive to growth factors such as HGF, EGF and TGF-α [32], [34].

Interruption of JNK/c-Jun and CyD1 signaling is potentially involved in the inhibition of liver regeneration in SFSLT. Also, CyD1 is an important target of the ERK and mTOR pathways in driving regeneration [35], [36]. According to our western blot analysis, CyD1 expression was barely detectable in sham-operated livers and FSG, but increased 18-fold in HSG. In TSG, however, CyD1 did not increase.

CyE plays a key role in liver regeneration and in CyE_1_ deficient mice the mitogenic response of hepatocytes to 70%-hepatectomy is impaired [37]. CyE was recently shown to be responsible for hepatocyte growth factor (HGF)-mediated G1/S progression in rat hepatocyte. primary culture via the proline-mTOR pathway [38].

As mentioned above, among growth factors, HGF is themost potent mitogen in mature hepatocytes and is known to enhance CyD1 expression, possibly via rapid activation of β-catenin. According to research from Kiyomasa Oka’s lab, HGF not only enhances CyD1 expression, but also up-regulates CyE_1_ to drive hepatocytes to enter S-phase. In this pathway, proline is necessary for enhancing ribosomal protein S6 (rpS6, mTOR specific target) phosphorylation, leading to the post-transcriptional up-regulation of CyE. According to our previous study on amphiregulin’s important role in stimulating liver regeneration in SFS transplantation, there is a marked increase in HGF mRNAs in TSG compared with FSG and HSG [20]. However, in the present study, mTOR and its downstream effector, p70S6K, as well as CyE, increased in HSG, but not in TSG. Similarly, CyD1 expression was paralleled closely by CyE expression (figure 7A), suggesting that a proline-mTOR-CyE pathway deficiency might be contributing to the suppressed regeneration of SFS grafts. Our finding that PCNA expression was markedly elevated in HSG, but barely detectable in TSG, lends further support to this notion. Similarly, parallel CyD1 and CyE expression indicates increasing regeneration in HSG and suppressed regeneration in TSG, with inhibited regeneration in small-for-size grafts unlikely due to a deficiency in pro-regenerative HGF.

A complex network of factors are involved in liver regeneration, accordingly, two pathways have been elucidated that are crucial: cytokine-dependent and cytokine-independent (growth factor-dependent) [39]. In the growth factor-dependent pathway, HGF and the epidermal growth Factor receptor (EGFR) ligand family are important growth factors that drive cell cycle progression during the process [40].

HGF binds to the c-Met receptor, leading to the activation of several downstream pathways including ERK 1/2, PI3K, S6 kinase and AKT. HGF/c-Met signaling is important in hepatoprotection from apoptosis and in the enhancement of hepatic repair after liver injury. Of all the signals participating in liver regeneration after partial hepatectomy, HGF signaling appears to be the most irreplaceable contributor [41]. In the present study, the activation of PI3K and its important downstream effector, Akt, which activates mTOR and p70S6K, were detected, showing a marked increase in HSG and inhibition in TSG (figure 7B). Similarly, ERK expression was also stimulated in HSG but suppressed in TSG. In addition, Akt has been proven important in the PPAR-β/PDK1/Akt pathway, and Inactivation of this pathway resulted in delayed liver regeneration in mice[42]. Ligands that bind to EGFR include EGF, TGF-α, heparin-binding EGF-like growth factor (HG-EGF) and amphiregulin (AR). EGFR also leads to the activation of the PI3K, ERK and JNK pathways. C-Jun-N-terminal kinase (JNK) activation, which is also preceded by the release of TNF-α, plays an important role in the interaction between the cytokine-dependent and growth factor-dependent pathways in liver regeneration. The growth factor + TNF-α→JNK→c-Jun phosphorylation→AP-1 activation→CyD1 upregulation pathway is critical in driving proliferation during liver regeneration. JNK blockade decreases survival after PH [43] and JNK2 leads to mitochondrial permeability transition (MPT), thus promoting graft injury. Graft survival is improved following transplantation of JNK2-deficient livers [19]. Of note, JNK blockade does not significantly increase apoptosis after PH, and in our study, JNK2 expression increased in HSG but was inhibited in TSG. The extent of apoptosis between the two grafts was not significantly different, allowing speculation that the JNK deficiency driving proliferation contributes more to suppressed regeneration after SFS liver transplantation.

In conclusion, suppressed liver regeneration is suspected to be the major factor contributing to graft dysfunction and failure after small-for-size liver transplantation. Inhibited proliferation and cell cycle pathway movement is suggested as a possible major cause, though the mechanisms remain to be elucidated. An increased understanding of the liver regeneration cascade in partial liver transplantation could lead to improved clinical outcomes from cadaveric-split or living-donor liver transplantation.

## Supporting Information

Figure S1. ARRIVE guidelines All the experimental design and procedure have followed the ARRIVE guidelines for animal research.
(PDF)
